# Shortened radiofrequency delivery time to optimize efficiency and safety of pulmonary vein isolation with the radiofrequency balloon: insights from the COLLABORATE registry

**DOI:** 10.1093/europace/euae227

**Published:** 2024-09-04

**Authors:** Alexandre Almorad, Domenico Giovanni Della Rocca, Alvise Del Monte, Johan Vijgen, Pieter Koopman, René Worck, Arne Johannessen, Caroline Lepièce, Antoine de Meester de Ravenstein, Teresa Strisciuglio, Sara Poggi, Giuseppe Stabile, Carmelo La Greca, Joseph Antoine Kheir, Laurence Jesel-Morel, Milad El Haddad, Amin Hossein, Charles Audiat, Roberto Scacciavillani, Luigi Pannone, Carlo de Asmundis, Gian-Battista Chierchia

**Affiliations:** Heart Rhythm Management Centre, Postgraduate Program in Cardiac Electrophysiology and Pacing, Universitair Ziekenhuis Brussel—Vrije Universiteit Brussel, Laarbeeklaan 101, 1090 Brussels, Belgium; Heart Rhythm Management Centre, Postgraduate Program in Cardiac Electrophysiology and Pacing, Universitair Ziekenhuis Brussel—Vrije Universiteit Brussel, Laarbeeklaan 101, 1090 Brussels, Belgium; Heart Rhythm Management Centre, Postgraduate Program in Cardiac Electrophysiology and Pacing, Universitair Ziekenhuis Brussel—Vrije Universiteit Brussel, Laarbeeklaan 101, 1090 Brussels, Belgium; Cardiology Department, Hartcentrum Hasselt, Jessa Ziekenhuis, Belgium; Cardiology Department, Hartcentrum Hasselt, Jessa Ziekenhuis, Belgium; Cardiology Lab, Gentofte Hospital, University of Copenhagen, Gentofte, Denmark; Cardiology Lab, Gentofte Hospital, University of Copenhagen, Gentofte, Denmark; Cardiology Department, CHU Helora Jolimont, Jolimont, Belgium; Cardiology Department, CHU Helora Jolimont, Jolimont, Belgium; Cardiology Department, Mediterranea Cardiocentro, Naples, Italy; Cardiology Department, University of Naples Federico II, Naples, Italy; Cardiology Department, Mediterranea Cardiocentro, Naples, Italy; Cardiology Department, Mediterranea Cardiocentro, Naples, Italy; Electrophysiology Unit, Fondazione Poliambulanza Istituto Ospedaliero, Brescia, Italy; Electrophysiology Unit, Fondazione Poliambulanza Istituto Ospedaliero, Brescia, Italy; Cardiology Department, Centre hospitalier regional de Strasbourg, Strasbourg, France; Independent Researcher, Helsinki, Finland; Independent Researcher, Brussels, Belgium; Heart Rhythm Management Centre, Postgraduate Program in Cardiac Electrophysiology and Pacing, Universitair Ziekenhuis Brussel—Vrije Universiteit Brussel, Laarbeeklaan 101, 1090 Brussels, Belgium; Heart Rhythm Management Centre, Postgraduate Program in Cardiac Electrophysiology and Pacing, Universitair Ziekenhuis Brussel—Vrije Universiteit Brussel, Laarbeeklaan 101, 1090 Brussels, Belgium; Heart Rhythm Management Centre, Postgraduate Program in Cardiac Electrophysiology and Pacing, Universitair Ziekenhuis Brussel—Vrije Universiteit Brussel, Laarbeeklaan 101, 1090 Brussels, Belgium; Heart Rhythm Management Centre, Postgraduate Program in Cardiac Electrophysiology and Pacing, Universitair Ziekenhuis Brussel—Vrije Universiteit Brussel, Laarbeeklaan 101, 1090 Brussels, Belgium; Heart Rhythm Management Centre, Postgraduate Program in Cardiac Electrophysiology and Pacing, Universitair Ziekenhuis Brussel—Vrije Universiteit Brussel, Laarbeeklaan 101, 1090 Brussels, Belgium

**Keywords:** Pulmonary vein isolation, Radiofrequency balloon, Atrial fibrillation, Shortened RF delivery, Oesophageal temperature

## Abstract

**Aims:**

Previous clinical studies on pulmonary vein isolation (PVI) with a radiofrequency balloon (RFB) reported safe and effective procedures using conventional ablation settings with 20/60 s RF delivery via posterior/anterior (PST/ANT) electrodes. The latest evidence suggests that reducing the application time to 15 s (s) on the posterior wall when facing the oesophageal region is as effective as applying 20 s. To prospectively assess whether reducing RF time on PST/ANT segments to 15/45 s can ensure sufficient quality of lesion metrics and compare the new shortened ablation settings with the conventional one in terms of safety, and effectiveness at 1-year.

**Methods and results:**

A total of 641 patients from seven European centres were enrolled in a collaborative registry, with 374 in the conventional RF delivery group and 267 in the shortened RF delivery group. Procedural outcomes, lesion metrics, and safety profiles were assessed and compared between the groups. Freedom of any atrial tachycarrythmias at one year was 85.4% and 88.2% in the SHRT and CONV groups, respectively. The shortened RF delivery strategy was associated with significantly shorter procedure times (median 63.5 vs. 96.5 min, *P* < 0.001) and shortened fluoroscopy exposure (median 10.0 vs. 14.0 min, *P* < 0.001) compared to conventional delivery. Efficacy metrics, including first-pass isolation rates and time to isolation, were comparable between groups. Shortened RF delivery was associated with a lower incidence of procedural complications (1.4% vs. 5.3%, *P* = 0.04) and optimized thermal characteristics.

**Conclusion:**

Analyses from the COLLABORATE registry demonstrate that shortening RF energy delivery times to 15/45 s (PST/ANT) during PVI with the RFB resulted in comparable freedom from recurrent atrial tachyarrhythmia compared to conventional delivery times with comparable efficiency and safety.

## Graphical Abstract

**Graphical Abstract:**
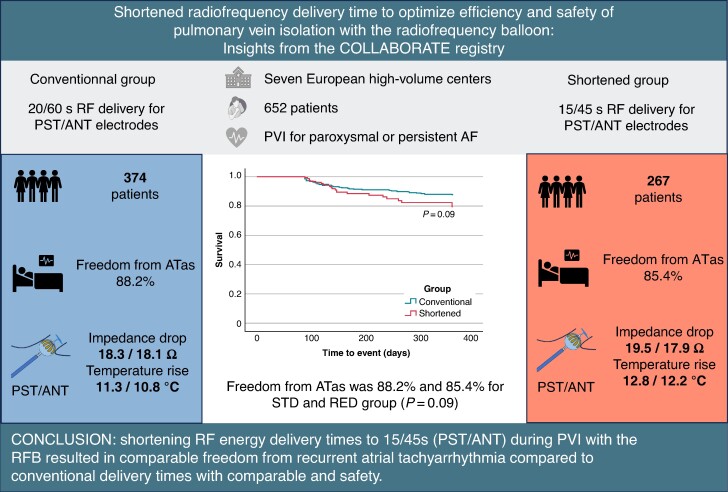

**What’s new?**

- Shortening RF energy delivery times to 15 s posteriorly and 45 s anteriorly during radiofrequency balloon catheter ablation for pulmonary vein isolation results in comparable freedom from recurrent atrial tachyarrhythmia when compared to conventional delivery times.
- The shortened RF delivery group and the conventional delivery group showed no compromise in procedural effectiveness.
- The shortened RF energy delivery time strategy was characterized by less oesophageal temperature rise than conventional delivery times.

## Introduction

Atrial fibrillation (AF) is the most prevalent cardiac arrhythmias, affecting millions of individuals around the globe.^1^ AF continues to impose a growing burden on healthcare systems globally, and the pursuit of interventions that not only address the complexity of this arrhythmia but also prioritize patient safety, procedural efficacy, and procedural efficiency has become paramount.^2^

Pulmonary vein isolation (PVI) is the cornerstone of AF ablation treatment.^3^ As the number of patients with AF requiring treatment continues to rise, single-shot technologies are emerging as the most effective solutions for addressing this growing need.^4^ Although pulsed-field ablation catheters are starting to emerge, the cryoballoon (CB) remains the prevailing technique. Recently a radiofrequency (RF) single-shot catheter was introduced, namely the radiofrequency balloon^5^ (RFB, Heliostar, Biosense Webster, CA, USA). The distinctiveness of the latter lies in its incorporation of RF technology, which sets it apart from other catheters and the ability for seamless integration with a 3D electroanatomical mapping system (CARTO®3, Biosense Webster, CA, USA). Compared to the commonly used single-shot technology, that is, CB, RFB appears to have a comparable profile regarding safety, efficacy, and efficiency but shows shorter dwell times and thermal delivery time.^6^

However, one of the main concerns that is often raised regarding the use of radiofrequency is the potential harm to adjacent structures, above all, the oesophagus and phrenic nerve. Several clinical studies have demonstrated the safety and efficacy of RFB.^7–9^ Recently, our group investigated and assessed the possibility of further enhancing RFB safety by reducing the time of RF energy delivery on electrodes facing the posterior (PST) wall without affecting lesion quality metrics.^10^ This study aimed to prospectively evaluate the safety and efficacy of shortened RF energy delivery time in both the posterior and anterior segments (15/45 s) compared with the conventional RF delivery time (20/60 s).

## Methods

### Study population

In this multicentre study conducted across seven European high-volume centres, 641 consecutive patients with paroxysmal and persistent AF scheduled for PVI using the RFB (Heliostar, Biosense Webster, Inc., Irvine, CA, USA) between January 2022 and November 2023 were enrolled. The study adhered to the ethical principles outlined in the Declaration of Helsinki (revised 2013 version) and was approved by the local ethics committees of the participating institutions.

### Ablation procedure

The patients were treated under general anaesthesia or deep/conscious sedation, and uninterrupted anticoagulation therapy. A circular multielectrode oesophageal temperature monitoring probe (CIRCA) was positioned to ensure complete coverage of the oesophageal path.

In all patients, a second diagnostic catheter, either a quadripolar or decapolar catheter, was introduced and positioned inside the coronary sinus to monitor atrial activity and allow superior vena cava (SVC) pacing during RF delivery in the right veins.

According to the operator’s preference, a single transseptal access was performed using a fixed sheath under transoesophageal echography guidance and/or fluoroscopy and a bolus of heparin was administered to reach and maintain an ACT level of 300–350 throughout the procedure. After exchanging the sheath with a dedicated deflectable one (14F, GUIDESTAR, OSCOR), the RFB and circular catheter (LassoStar, Biosense Webster, CA, USA) were introduced into the left atrium (LA). An electroanatomical map was created using LassoStar.

As described previously,^6^ the RFB was carefully positioned at the PV ostias. Before starting RF delivery, operators were asked to identify and select the generator electrodes facing the posterior wall (PST). A minimum of three PST electrodes were selected in all patients to guarantee a shorter ablation time along the entire length of the posterior wall. The irrigation flow rate was 35 mL/min during RF energy delivery (5 mL/min when RF was off).

The oesophageal temperature was monitored during ablation. In the case of an oesophageal temperature rise (>2°C) from baseline, the PST electrodes were manually switched off. If the temperature continued to increase, the electrodes adjacent to the PST electrodes were switched off.

Before ablating, the ostia of each of the right PVs proximity to the phrenic nerve was checked by pacing the anterior electrodes at 10 mA for 2 ms. In case of capture, the RFB was repositioned and the phrenic nerve capture test was repeated. CS catheter was always positioned inside the SVC, and continuous pacing at maximum output was performed to ensure phrenic nerve capture during energy delivery.

During the year 2022 (January–December), conventional radiofrequency (RF) time settings were used. Specifically, the radiofrequency ablation duration in the posterior (PST) and the anterior (ANT) electrodes was set to 20 and 60 s, respectively. Subsequently, starting from January 2023, the ablation settings were changed to 15 and 45 s for the PST and ANT electrodes, respectively. Patients receiving ablation with conventional time settings were categorized as the ‘CONVENTIONAL’ group (CONV), whereas those treated with shorter ablation settings were classified as the ‘SHORTENED’ group (SHRT; *Table 1*).

**Table 1:** Radiofrequency ablation application times and patient group classifications

Electrodes	Shortened RF delivery time	Conventional RF delivery time	Power/temperature target
PST	15 s	20 s	15 W/55°
ANT	45 s	60 s	15 W/55°

PST, posterior electrodes; ANT, anterior electrodes.

During ablation, PV potentials were monitored using a circular diagnostic catheter to evaluate single-shot and real-time to isolation (TTI).

Electrodes temperature (*T*) and impedance (*Z*) values were measured over the entire tissue–electrode interface.

### Procedure endpoints

As defined previously,^6^ single-shot isolation was time to isolation of <12 s. In cases of a longer time to isolation (TTI), an extra application, segmental or circumferential, was achieved.

Acute isolation was defined as confirmed PVI validated with a multipolar catheter at the end of the procedure, and waiting time/adenosine proof was left at the operator’s discretion.

The skin-to-skin time was defined as the time from the first puncture to the withdrawal of the last catheter. Dwell time was defined as RFB time spent in the LA, while RF time was defined as the time of effective energy delivery.

### Oesophageal temperature monitoring

All centres participating in this study used the same oesophageal multisensor temperature probe (CIRCA multisensory, Abbott), with the oesophageal temperature (ET) alarm set to 39°C. Baseline ET and ET_max_ values were systematically recorded. According to the physician’s preference, prophylactic proton pump inhibitors (PPI) were administered to patients for up to four weeks post-ablation, as per the Heart Rhythm Society consensus.^4^ As per protocol, no mandated gastroscopy was specified in patients with high ET, nevertheless a control could be carried out if the physician deemed it necessary.

### Off-line analysis of lesion metrics

The metrics of all lesions, including the duration of RF delivery, *Z*, and *T* for each posterior and anterior electrode, were extracted from the generator for off-line analyses.

We defined the plateau of temperature *T*(t) = *T*_100%_ or impedance *Z*(t) = *Z*_100%,_ where *T*_100%_ and *Z*_100%_ were calculated based on the 95% quantile of the temperature or impedance values throughout the ablation session.^10^ This plateau corresponds to the first instance when these values show no significant temporal variations until the end of the ablation session. We collected the following parameters for each posterior electrode: baseline impedance (*Z*_base_) and temperature (*T*_base_), impedance at the plateau (*Z*_100%_) and its 95th (*Z*_95%_) percentile values, and temperature at the plateau (*T*_100%_) and its 95th (*T*_95%_) percentile values. The impedance drop at the plateau (Δ*Z*_100%_) and the temperature rise to reach the plateau (Δ*T*_100_) were also computed with their 95th (Δ*Z*_95%_; Δ*T*_95%_) percentiles. The time required to reach each value was calculated for each electrode. All values are reported as medians (Q1–Q3).

### Post-procedural management and follow-up

All patients underwent continuous telemetry monitoring for at least 24 h after the procedure and were discharged after overnight observation if no complications occurred. Oral anticoagulation was started the same evening after ablation and continued for at least two months; thereafter, it was prolonged according to the patient’s thromboembolic risk profile. Antiarrhythmic drugs (AAD) were discontinued at the latest three months after ablation for paroxysmal AF.

We also collected any major periprocedural complications [e.g. death, atrioesophageal fistula, stroke/transient ischaemic attack (TIA), pericardial effusion/tamponade with/without surgical treatment, myocardial infarction, and persistent phrenic palsy] occurring within 7-day post-procedure (except for atrioesophageal fistula). Minor complications have also been reported, including vascular access complications requiring treatment, pericarditis, and transient phrenic palsy.

The clinical follow-up strategy included at least three in-person outpatient visits at 3, 6, and 12 months post-ablation. Each visit included a clinical examination and 12-lead electrocardiogram. Furthermore, at least one 24 h Holter monitor was recorded during the first 12 months post-procedure. Regular telephone consultations were conducted between scheduled visits.

### Study endpoints

This study aimed to compare the efficacy and safety outcomes of PVI using RFB between the CONV and SHRT groups.

Efficacy outcomes included single-shot isolation rate, time to isolation (TTI), acute isolation rate, skin-to-skin time, dwell time, fluoroscopy time, and absence of any atrial tachyarrhythmias (ATas) > 30 s during follow-up.

The safety endpoints included the occurrence of adverse events within 30 days of the ablation procedure and the occurrence of an oesophageal temperature rise (OTR) during ablation. The adverse events included cardiac tamponade, diaphragmatic paralysis, stroke, death, heart block, and myocardial infarction.

### Analysis and statistics

The results are presented as absolute values with percentages, medians, and interquartile ranges. Normally and non-normally distributed continuous variables were compared using the Student’s *t*-test and Mann–Whitney *U* test, respectively, whereas categorical variables were compared using the χ^2^ test. The Kaplan–Meier estimator, the product limit estimator, was used to estimate and plot the survival functions, and time-to-event analysis was performed using the log-rank test (Mantel–Cox test) in all patients exceeding 6 months of follow-up. A two-sided α of <.05 was considered statistically significant. All statistical analyses were performed using SPSS (Statistical Package for the Social Sciences), version 27.0 software (IBM SPSS Statistics), and GraphPad Prism version 10.2.2 (GraphPad Software, Boston, MA, USA).

## Results

### Clinical characteristics

Between January 2022 and November 2023, 641 patients were enrolled in eight European centres, 374 with the CONV protocol and 267 with the SHRT protocol. Five hundred nineteen patients met the follow-up criteria and were included in the survival analysis: 348 with the CONV protocol and 171 with the SHRT protocol. The two groups were comparable in terms of baseline characteristics, except for the distribution of coronary artery disease (15.6% vs. 22.1%, *P* < 0.05) (*Table 2*).

**Table 2:** Baseline demographic and clinical characteristics

Characteristics	CONVENTIONAL	SHORTENED	*P*-value
Number of patients, *n*	374	267	–
Age (years)	65.5 (58.0–74.0)	67.0 (60.0–73.0)	0.8
BMI (kg/m^2^)	27.4 (24.7–31.1)	27.3 (24.5–31.1)	0.7
Male, *n* (%)	234 (62.8)	181 (67.8)	0.21
Type of AF, *n* (%)			0.07
Paroxysmal, *n* (%)	234 (62.6)	139 (70.6)	
Persistent, *n* (%)	140 (37.4)	58 (29.4)	
Left atrial diameter (mm)	39.0 (31.0–45.0)	37.0 (30.0–43.0)	0.3
Left ventricular ejection fraction (%)	55.0 (55.0–60.0)	55.0 (55.0–60.0)	0.9
CHA2DS2-VASc score	2 (1–4)	2 (1–3)	0.74
Hypertension, *n* (%)	223 (59.6)	150 (56.4)	0.42
Diabetes mellitus, *n* (%)	58 (15.5)	49 (18.3)	0.39
Coronary artery disease, *n* (%)	58 (15.6%)	59 (22.1%)	0.04
Congestive heart failure, *n* (%)	70 (18.7)	36 (13.5)	0.09
Stroke/transient ischaemic attack, *n* (%)	40 (10.7)	28 (10.5)	1
Chronic kidney disease, *n* (%)	63 (17.0)	33 (12.4)	0.12

### Procedural details lesion metrics and effectiveness

The median procedure time was 96.5 (71–129) vs. 63.5 (50.0–82.7) min (*P* < 0001), with a median dwell time of 30.0 [19.0; 45.5] vs. 28.0 [19.0; 40.0] min (*P* = 0.24) and a median fluoroscopy time of 14.0 [7.0; 21.0] vs. 10.0 [5.0; 16.7] min (*P* < 0.0001) in the CONV and SHRT groups, respectively. There was no difference in first-pass isolation between the CONV group, with 3908 (91.7%) veins vs. 649 (90.7%) in the SHRT group. In addition, the TTI was comparable for all veins in both groups. Furthermore, in cases where more than one application per PV was required, the total number of applications for each PV was similar between the two groups. Moreover, a left common ostium was present in 9.3% and 18.2% (*P* < 0.005) of patients in the CONV and SHRT groups, respectively. The complete procedural characteristics of both groups are summarized in *Table 3*. No major periprocedural complications occurred in either of the groups (*Table 4*). Peripheral vascular access haematoma was observed in five patients in the CONV group and three in the SHRT group (*P* = 0.81), whereas uncomplicated pericarditis occurred in three and zero patients, respectively (*P* = 0.1). Phrenic nerve capture was transiently lost in seven patients in the CONV group and one in the SHRT group (*P* = 0.03). All patients recovered phrenic nerve function at the 1-month follow-up visit.

**Table 3:** Procedure outcomes between the two groups

Procedure outcomes	CONVENTIONAL	SHORTENED	*P*-value
Procedure time (skin to skin), min	96.5 (71–129)	63.5 (50.0–82.7)	<0.001
Total PVs target for ablation	1318	716	–
Left common PV, *n* (%)	31	34	0.005
Median time to isolation overall (s)	9.5 (8.0–11.5)	9.5 (8.0–11.0)	–
Median time to isolation by veins (s)			
Left superior PV	10.0 (9.0–12.0)	10.0 (8.0–12.0)	0.25
Left inferior PV	9.0 (8.0–11.0)	9.0 (7.2–11.0)	0.7
Right inferior PV	9.0 (8.0–11.0)	9.0 (8.0–11.0)	0.47
Right superior PV	9.0 (8.0–10.0)	9.0 (8.0–10.0)	0.73
Single-shot isolation by veins, *n* (%)			
Left superior PV	299 (90.1)	160 (87.9)	0.46
Left inferior PV	316 (96.1)	165 (93.8)	0.27
Right inferior PV	293 (89.3)	162 (90.5)	0.76
Right superior PV	300 (91.2)	162 (90.5)	0.87
Fluoroscopy time, min	14.0 (7.0–21.0)	10.0 (5.0–16.7)	<0.0001
LA dwelling time, min	30.0 (19.0–45.5)	28.0 (19.0–40.0)	0.24
AAD at discharge, *n* (%)	202 (60.3)	169 (66.5)	0.12
AAD after 1 year follow-up, *n* (%)	30 (7.9)	39 (14.5)	0.009
ATas after 1 year follow-up, *n* (%)	41 (14.5)	25 (7.7)	0.086

AAD, antiarrhythmic drugs; ATas, atrial tachyarrhythmias; LIPV, left inferior pulmonary vein; LSPV, left superior pulmonary vein; RIPV, right inferior pulmonary vein; RSPV, right superior pulmonary vein; TTI, time to isolation.

**Table 4:** Complications

	CONVENTIONAL	SHORTENED	*P*-value
Global	20 (5.3)	4 (1.4)	0.04
Major	0 (0.0)	0 (0.0)	–
Pericarditis with conservative treatment	3 (0.8)	0 (0.0)	–
Vascular aneurysm with conservative treatment	2 (0.5)	0	–
Vascular access haematoma	6 (1.6)	3 (1.0)	–
Tamponade	2 (0.5)	0 (0.0)	–
Phrenic nerve injury			
Permanent	0 (0.0)	0 (0.0)	–
Transient	7 (1.8)	1 (0.3)	0.03

CONV settings counted 4162/7720 electrodes for PST/ANT electrodes, while the SHRT group counted 2199/3280 electrodes for PST/ANT electrodes.

**Figure 1:**
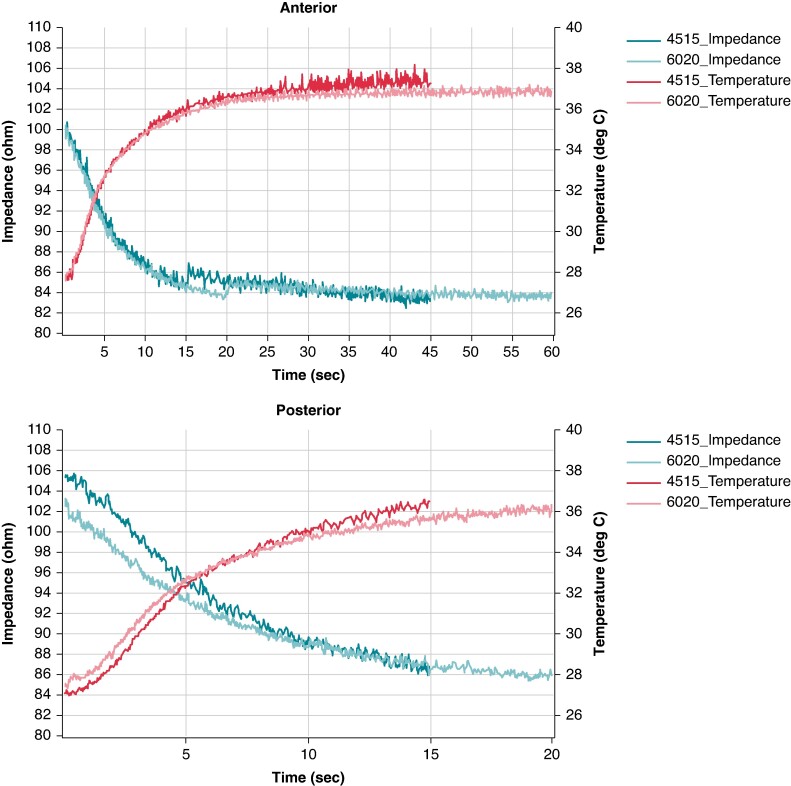
Time evolution of median impedance drop (left axis) and temperature rise (right axis) for the CONV and SHRT groups for posterior (*A*) and anterior (*B*) electrodes.

As depicted in *Figure 1*, the median electrodes’ impedance drop for PST electrodes was smaller in the CONV group than in the SHRT group (18.3 (12.6–24.6) vs. 19.48 (13.9–25.1) Ω, *P* < 0.001). In addition, higher temperature rise was observed in the CONV vs. SHRT group (11.35 (7.4–15.2) °C vs. 10.80 (7.4–13.9) °C, *P* < 0.001).

The median impedance drop did not differ between CONV and SHRT ANT electrodes. Meanwhile, a higher temperature rise was observed in the CONV group than in the SHRT group (12.80 (7.9–18) °C vs. 12.20 (8–16.7) °C, *P* < 0.001). The complete lesion metric value comparison for both groups is summarized in *Table 5*.

**Table 5:** Comparison of lesion metric values between CONV and SHRT groups after radiofrequency delivery

Groups		95%			Plateau		
		STA	SHRT	*P*-value	STA	SHRT	*P*-value
PST	Time to impedance	11.4	9.8	0.0001	13.2	11.0	0.0001
		(8.9–13.5)	(8.4–10.9)		(10.3–15.6)	(9.4–12.2)	
	Delta impedance	17.4	18.5 (13.2–23.8)	0.0001	18.3	19.5	0.0001
		(12.0–23.6)			(12.6–24.8)	(13.9–25.1)	
	Time to temperature	10.2	9.3	0.0001	16.4	13.3	0.0001
		(6.7–13.2)	(6.9–10.8)		(12.9–18.6)	(11.6–14.4)	
	Delta temperature	9.4	8.9	0.0001	11.3	10.8	0.0001
		(5.6–13.1)	(5.7–11.9)		(7.4–15.2)	(7.4–13.9)	
ANT	Time to impedance	12.7	11.8	0.0001	15.1	13.9	0.0001
		(8.9–19.2)	(8.7–20.2)		(10.3–29.8)	(10.0–27.3)	
	Delta impedance	17.2	17.0	0.16	18.1	17.9	0.16
		(12.4–22.9)	(11.9–22.8)		(13.1–24.1)	(12.5–24.0)	
	Time to temperature	14.8	13.8	0.0001	30.1	28.87	0.0001
		(8.1–24.4)	(8.1–21.0)		(18.4–47.3)	(18.0–38.3)	
	Delta temperature	10.8	10.2	0.0001	12.8	12.2	0.0001
		(6.1–15.7)	(6.1–14.5)		(7.9–18.0)	(8.0–16.7)	

Comparison of delta impedance drop and delta temperature rise at each electrode from baseline to total RF delivery between the groups. The time to 95% of the plateau is also shown.

The baseline impedance of the PST electrodes was lower in the CONV group than in the SHRT group, respectively 102.6 (96.0–111.0) Ω vs. 105.6 (97.9–114.7) Ω. In contrast, the temperature baseline was higher, respectively 27.6 (26.3–29.2) °C and 27.2 (26.2–28.7) °C (*P* < 0.001). None of the groups showed any differences in baseline impedance or temperature for the ANT electrodes. This information is summarized in Table A, attached to Annex A.

### Follow-up outcomes

**Figure 2:**
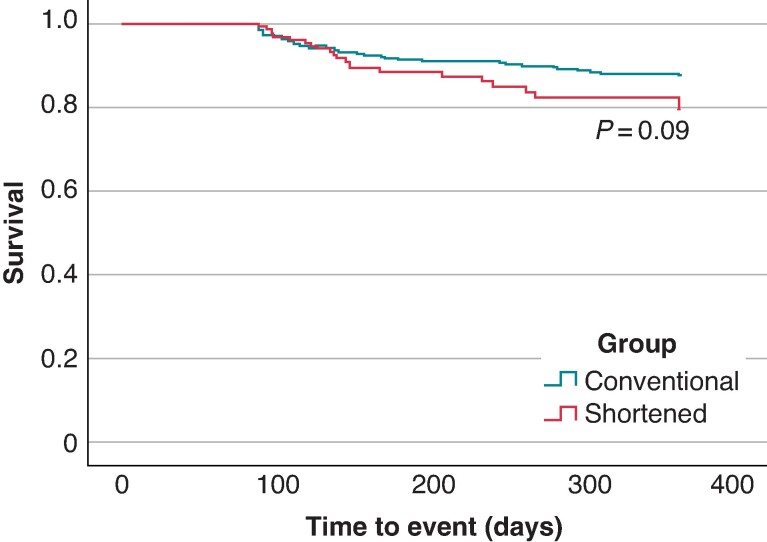
Kaplan–Meier curve of any atrial tachyarrhythmias (ATas)-free survival during the follow-up; the freedom from ATas was 88.2% and 85.4% for CONV and SHRT groups, respectively.

Five hundred nineteen patients out of AAD (348 in CONV and 171 in SHRT) fulfilled the follow-up protocol and were included in the survival analyses. With a median follow-up of 357.0 (219–443) days, the ATas-free rates were 88.2% and 85.4% in the CONV and SHRT groups, respectively (*P* > 0.05; *Figure 2*).

In the CONV group, among the patients with at least one lesion with an OTR > 39°C, OTR > 41°C occurred in 60 (72.3%) patients, whereas in the SHRT group, it occurred less often in 32 (50.8%) patients (*P* < 0.01). Gastroscopy was performed in 53 patients, four of them, all from the CONV group, showed a limited erythema empirically treated with PPI.

## Discussion

The study analysis highlights that a shorter RF delivery time with the RFB optimizes the efficiency with comparable thermal characteristics and improves the safety profile of PVI. This study can be summarized as follows: (1) shortening RF energy delivery times during radiofrequency balloon catheter ablation for PVI resulted in comparable freedom from recurrent atrial tachyarrhythmia when compared to conventional delivery times; (2) efficacy parameters, including lesion metrics, first-pass isolation rates, and time to isolation, were comparable between the shortened RF delivery group and the conventional delivery group, demonstrating no compromise in procedural effectiveness; (3) the shortened RF energy delivery time strategy was characterized by shorter procedure times and fluoroscopy exposure, with less oesophageal temperature rise compared to conventional delivery times; and (4) the shortened RF delivery strategy was associated with a lower incidence of procedural complications.

### Long-term outcomes and follow-up

Long-term follow-up data revealed comparable rates of atrial tachyarrhythmia (ATas)-free survival between the conventional and shortened RF delivery groups. Indeed, at a follow-up of 11.7 (7.2–14.6) months, the overall freedom from ATas recurrence out of AAD was identical in both groups (88.2% for CONV and 85.4% for SHRT, *P* > 0.05), with no difference in recurrence type. These results are consistent with those of recent studies that investigated various ablation techniques. For instance, the RADIANCE study, which first evaluated the RFB, reported an ATas freedom rate at 12 months of 86.4% in a cohort of 37 patients with paroxysmal AF.^7^ Similarly, the FIRE AND ICE trial, the largest randomized controlled trial comparing PVI with cryoballoon (CB) and point-by-point RF in paroxysmal AF, demonstrated comparable efficacy.^11^ In various studies, ATas freedom rate at 12 months reported ranged from 78.2% to 87%,^12–16^ Other recent technologies, such as pulsed-field ablation (PFA), have found similar results (84.5%).^17^

These findings suggest that optimizing energy delivery parameters during ablation does not compromise the durability of PVI, as evidenced by sustained rhythm control over extended follow-up periods.

### Procedural outcomes and lesion metrics

Pulmonary vein isolation represents a fundamental approach in AF ablation therapy. As the demand for AF treatment increases, there is a notable shift towards more time efficient techniques, particularly those employing single-shot technologies. Cryoballoon has emerged as the dominant method,^4^ yet the RFB distinguishes itself by utilizing radiofrequency energy instead of cryoablation and integrating it with a 3D electroanatomical mapping system.^12,13,18,19^ Compared with the established single-shot cryoballoon approach, RFB demonstrates comparable safety, efficacy, and efficiency metrics, but with shortened dwell and thermal delivery times.^6,18^

Nevertheless, concerns persist regarding potential radiofrequency-related injuries to adjacent structures, particularly the oesophagus. Our recent investigation focused on optimizing RFB safety by reducing the duration of RF energy delivery on electrodes targeting the posterior (PST) wall while maintaining high-quality lesion metrics.^20^

The results of this study indicate that reducing RF energy delivery times from 20/60 s to 15/45 s for PST/ANT electrodes, respectively, during PVI procedures significantly shortened the total procedure duration and fluoroscopy exposure without compromising procedural efficacy, from 96.5 to 63.5 min and 14.0 to 10.0 min (*P* < 0.05), respectively. The procedure duration in the CONV group was similar to the first multicentric reports on RFB, where RADIANCE and SHINE studies reported 102 and 88 min, respectively.^7,8^ Moreover, it is similar to those reported in other multicentric studies regardless of the energy source: 80.6–131.7 min for CB and 76–151 min for RFA.^9,19,21^ However, this is longer than the procedure time reported in more recent studies (46–77 min).^6^ The latter finding aligns with the procedure time in the SHRT group. It is reasonable to assume that the shortened time in the SHRT group in this study was mainly due to the operator’s growing experience with the catheter. The CONV group fluoroscopy time aligns with the literature-reported value of 7–21 min, while the SHRT group fluoroscopy time was 30% shorter, 5–16 min. Similarly to procedure time reduction, this might be partly due by the difference between the CONV and SHRT protocols but mainly by the operators’ learning curve. By gaining confidence in the system over time, operators tend to rely more on the 3D mapping projections and less on the fluoroscopy. Noteworthy, inclusion in both groups were separated by a period of 12 months. We contend that this duration provides ample opportunity to become proficient with a new catheter, refine techniques, and optimize the ablation workflow. More than the 15 s difference in RF delivery, ultimately, learning curve resulted in shorter procedure times and reduced fluoroscopy time.

First-pass isolation showed a comparably high rate across all PVs, with a mean of 91.7% for the CONV group and 90.7% for the SHRT group, and a median time to isolation of 9.5 (8.0–11.5) and 9.5 (8.0–11.0), respectively.

Despite the reduction in procedural duration, key efficacy metrics, such as first-pass isolation rates and time to isolation, remained comparable between the conventional and shortened RF delivery groups and comparable to values reported by other trials ^8,22^ This finding aligns with studies like the one by Reddy *et al*.^23^, which highlighted the importance of maintaining effective lesion creation parameters while optimizing energy delivery times. These results are also consistent with data on the cryoballoon^11,13^ and PFA.^17,24,25^ The concordance in efficacy metrics suggests that shorter RF energy delivery times do not necessarily compromise the primary endpoint of PVI, which is an essential aspect of successful AF ablation.

### Lesion metrics and thermal characteristics

Previous studies have recommended achieving post-ablation impedance drops exceeding 12 Ω, and temperature increases >6°C.^7,26,27^ Del Monte *et al*.,^27^ in a recent publication, suggested that achieving an impedance drop exceeding 19.2 Ω and a temperature rise exceeding 11.1°C may serve as potential predictors of acute, persistent single-shot isolation. However, the analysis did not differentiate between posterior and non-posterior electrode effects. Given the thinner tissue composition of the left atrium–pulmonary vein (LA–PV) junction posteriorly compared to other anatomical segments,^28,29^ it is prudent to target lower impedance drops and temperature rises to safeguard adjacent structures while still achieving acute and durable lesions.^30–32^

Detailed analysis of lesion metrics is discussed in the Appendix. In short, given the significant number of electrodes that demonstrated outstanding lesion metrics, including a substantial drop in impedance (>12 Ω) and a notable increase in temperature (more than 6°C), it appears reasonable to explore the possibility of reducing the RF delivery time for both anterior and posterior electrodes.

The observed differences in impedance and temperature profiles underscore the potential for more tailored energy delivery strategies to optimize lesion formation while minimizing collateral tissue damage, eventually combining with other energy sources like pulsed-field energy.

### Safety profiles and complications

Notably, this prospective large multicentre study encountered no major complications across all groups, including pericardial effusion, stroke, TIA, atrial–oesophageal fistulas, or pulmonary vein (PV) stenosis. Importantly, adopting a shortened RF delivery strategy was associated with a decreased incidence of minor procedural complications, encompassing global issues such as pericarditis, vascular complications, and phrenic nerve injury.

Regarding minor complications, transient phrenic nerve injury occurred more frequently in the conventional (CONV) group than in the shortened (SHRT) group (seven vs. one patient, *P* = 0.03). These findings are consistent with previous reports.^7–9^ It is also plausible that growing experience with the RFB may have improved its safety profile. Importantly, all phrenic nerve injuries resolved during follow-up visits without requiring additional treatment.

Our study observed a significantly lower oesophageal temperature rise (OTR) in the shortened RF delivery group than that in the conventional group. Specifically, a higher proportion of patients in the conventional group experienced OTR exceeding 41°C than those in the shortened group (72.2% vs. 50.8%, *P* < 0.01). This translated with a limited number of patients with oesophageal erythema treated with pump inhibitors. Notably, all of them where in the CONV group, suggesting a better safety profile with shorter RF delivery time posteriorly, as suggested by a previous multicentric report.^20^ Literature suggests that minimizing oesophageal thermal injury during AF ablation procedures may reduce the risk of serious complications, such as oesophageal injury, atrial–oesophageal fistulas, or other gastrointestinal complications.^33,34^ Although direct evidence linking shortened OTR to long-term complication reduction is limited, it is plausible that mitigating oesophageal heating could translate into improved patient outcomes and decreased adverse events.

### Limitations

This study has several limitations. This was a non-randomized clinical study; nevertheless, this multicentre prospective registry including consecutive patients reflects real-life data. More extensive studies are required to evaluate the generalizability of these results. On the free survival curve, a trend of higher recurrence rate appears in the SHRT group. However, only a longer follow-up duration accurately determines the true recurrence rate. Differences in operator experience across the participating centres may have influenced the procedural outcomes and complication rates. No mandated gastroscopy was scheduled in patient with OTR, making it difficult to assess the safety of the RFB in this particular group of patients.

### Clinical implications and future directions

Our study contributes valuable insights into the optimization of RF energy delivery strategies for AF ablation using an RF balloon. This highlights the potential of shortened energy delivery times to enhance procedural efficiency and safety. Moreover, the efficacy of the RFB could potentially be further enhanced by integrating additional technologies, such as a pulsed-field energy source. This combination holds promise for optimizing lesion creation and procedural outcomes in atrial fibrillation ablation.

## Conclusions

Analyses from the COLLABORATE registry demonstrate that shortening RF energy delivery times to 15/45 s (PST/ANT) during PVI with the RFB resulted in comparable freedom from recurrent atrial tachyarrhythmia compared to conventional delivery times with comparable and safety.

## Data Availability

The data underlying this article will be shared on reasonable request to the corresponding author.

## Appendix

### Annex A

For PST electrodes, impedance plateaus of 84.6 (79.3–90.2) Ω vs. 86.5 (81.6–92.2) (*P* < 0.0001) were reached after 13.2 (10.3–15.6) vs. 11.0 (9.4–12.2) s (*P* < 0.0001) for CONV and SHRT, respectively. Temperature plateaus of 38.7 (35.8–42.3) vs. 37.9 (35.5–40.8) °C (*P* < 0.0001) after 16.4 (12.9–18.6) vs. 13.3 (11.6–14.4) s (*P* < 0.0001) for CONV and SHRT, respectively (Table A). For ANT electrodes, impedance plateaus of 81.3 (76.2–87.0) Ω vs. 81.9 (77.0–87.4) (*P* > 0.05) were reached after 15.1 (10.3–29.8) vs. 13.9 (10.0–27.3) s (*P* < 0.0001) for CONV and SHRT, respectively. Temperature plateaus of 40.3 (37.0–44.8) vs. 39.7 (37.0–43.9) °C (*P* < 0.0001) after 30.1 (18.4–47.3) vs. 28.87 (18.0–38.3) s (*P* < 0.0001) for CONV and SHRT, respectively (*Tables 4*). Plateaus and time to reach ΔZ_95%_ are also presented in *Table 5* and follow the same trend for PST and ANT electrodes.

**Table A:** Comparison of lesion metric values between CONV and SHRT groups after radiofrequency delivery

Groups		Baseline			95%			Plateau		
		STA	SHRT	*P*-value	STA	SHRT	*P*-value	STA	SHRT	*P*-value
PST	Time to impedance	–	–	–	11.4 (8.9–13.5)	9.8 (8.4–10.9)	0.0001	13.2 (10.3–15.6)	11.0 (9.4–12.2)	0.0001
	Impedance	102.6 (96.0–111.0)	105.6 (97.9–114.7)	0.0001	85.6 (80.3–91.0)	87.5 (82.6–93.2)	0.0001	84.6 (79.3–90.2)	86.5 (81.6–92.2)	0.0001
	Time to temperature	–	–	–	10.2 (6.7–13.2)	9.3 (6.9–10.8)	0.0001	16.4 (12.9–18.6)	13.3 (11.6–14.4)	0.0001
	Temperature	27.6 (26.3–29.2)	27.2 (26.2–28.7)	0.0001	36.8 (34.0–40.2)	36.0 (33.7–38.8)	0.0001	38.7 (35.8–42.3)	37.9 (35.5–40.8)	0.0001
ANT	Time to impedance	–	–	–	12.7 (8.9–19.2)	11.8 (8.7–20.2)	0.0001	15.1 (10.3–29.8)	13.9 (10.0–27.3)	0.0001
	Impedance	99.7 (93.2–107.4)	99.8 (92.8–108.0)	0.7	82.3 (77.2–87.9)	82.8 (77.9–88.4)	0.16	81.3 (76.2–87.0)	81.9 (77.0–87.4)	0.16
	Time to temperature	–	–	–	14.8 (8.1–24.4)	13.8 (8.1–21.0)	0.0001	30.1 (18.4–47.3)	28.87 (18.0–38.3)	0.0001
	Temperature	28.0 (26.4–29.6)	27.8 (26.5–30.0)	0.5	38.3 (35.1–42.6)	37.2 (35.1–41.7)	0.0001	40.3 (37.0–44.8)	39.7 (37.0–43.9)	0.0001

Comparison of impedance drop and temperature rise at each electrode from baseline to total RF delivery between groups. The time to 95 of the plateau is also shown. Our analysis revealed distinct differences in impedance and temperature profiles between the conventional and shortened RF delivery groups for the PST and ANT electrodes. Notably, the shortened RF delivery approach was associated with larger impedance drops and a lower temperature rise for the PST electrodes, while only showing a shortened temperature rise with no difference in impedance drops for the ANT electrodes. For the PST electrodes, we have shown in a previous study that 17.5 Ω impedance drops were reached after 11.8 s; on the other hand, the temperature increased by 9.1°C after 10.1 s.^10^ In the current study on larger number of patients, we found that a 19.5 (13.9–25.1) Ω impedance drop was reached after 11.0 (9.4–12.2) s, and it was already 18.5 (13.2–23.8) Ω after 9.8 (8.4–10.9) s. On the other hand, the temperature achieved 10.8 (7.4–13.9) °C to reach a plateau after just 13.3 (11.6–14.4) s, and 8.9 (5.7–11.9) °C after 9.3 (6.9–10.8) s. In other words, not only does the SHRT group confirm that a shortened time of application of PST electrodes seems to achieve sufficient lesions, but an even shorter RF application of ∼12 s could be possible. In addition, this study reports the lesion metrics for the anterior electrodes. In the SHRT group, our lesion metric analysis of RF delivery showed that a 17.9 (12.5–24.0) Ω impedance drop was reached after 13.9 (10.0–27.3) s, and it was already 17.0 (11.9–22.8) Ω after 11.8 (8.7–20.2) s. On the other hand, the temperature achieved 12.2 (8.0–16.7) °C of its increase to reach the plateau after 28.9 (18.0–38.3) s, and 10.2 (6.1–14.5) °C after 13.8 (8.1–21.0) s. The CONV group showed very similar results, except for the temperature rise, which was higher: 30.1 (18.4–47.3) at its plateau after 30.1 (18.4–47.3) s. Consequently, decreasing the RF duration from 60 to 45 s for the ANT electrode maintained the same lesion profile with a significant beneficial time.
